# Estimating the Neutralizing Effect and Titer Correlation of Semi-Quantitative Anti-SARS-CoV-2 Antibody Immunoassays

**DOI:** 10.3389/fcimb.2022.822599

**Published:** 2022-04-14

**Authors:** Beomki Lee, Jae-Hoon Ko, Jiho Park, Hee-Won Moon, Jin Yang Baek, Sunhee Jung, Hee-Young Lim, Kyung-Chang Kim, Kyungmin Huh, Sun Young Cho, Cheol-In Kang, Doo Ryeon Chung, Hee Jae Huh, Chi Ryang Chung, Yae-Jean Kim, Eun-Jeong Joo, Eun-Suk Kang, Kyong Ran Peck

**Affiliations:** ^1^ Department of Laboratory Medicine and Genetics, Samsung Medical Center, Sungkyunkwan University School of Medicine, Seoul, South Korea; ^2^ Division of Infectious Diseases, Department of Medicine, Samsung Medical Center, Sungkyunkwan University School of Medicine, Seoul, South Korea; ^3^ Division of Infectious Diseases, Department of Medicine, Konkuk University Medical Center, Konkuk University School of Medicine, Seoul, South Korea; ^4^ Department of Laboratory Medicine, Konkuk University Medical Center, Konkuk University School of Medicine, Seoul, South Korea; ^5^ Asia Pacific Foundation for Infectious Diseases (APFID), Seoul, South Korea; ^6^ Division of Emerging Virus and Vector Research, National Institute of Health, Korea Disease Control and Prevention Agency, Cheongju, South Korea; ^7^ Department of Critical Care Medicine, Samsung Medical Center, Sungkyunkwan University School of Medicine, Seoul, South Korea; ^8^ Division of Infectious Diseases and Immunodeficiency, Department of Pediatrics, Samsung Medical Center, Sungkyunkwan University School of Medicine, Seoul, South Korea; ^9^ Division of Infectious Diseases, Department of Medicine, Kangbuk Samsung Hospital, Sungkyunkwan University School of Medicine, Seoul, South Korea

**Keywords:** SARS-CoV-2, COVID-19, serology, immunoassay, antibody responses, neutralizing antibody, spike (S) protein, nucleopcapsid (NP) protein

## Abstract

For the clinical application of semi-quantitative anti-SARS-CoV-2 antibody tests, the analytical performance and titer correlation of the plaque reduction neutralization test (PRNT) need to be investigated. We evaluated the analytical performance and PRNT titer-correlation of one surrogate virus neutralization test (sVNT) kit and three chemiluminescent assays. We measured the total antibodies for the receptor-binding domain (RBD) of the spike protein, total antibodies for the nucleocapsid protein (NP), and IgG antibodies for the RBD. All three chemiluminescent assays showed high analytical performance for the detection of SARS-CoV-2 infection, with a sensitivity ≥ 98% and specificity ≥ 99%; those of the sVNT were slightly lower. The representativeness of the neutralizing activity of PRNT ND_50_ ≥ 20 was comparable among the four immunoassays (Cohen’s kappa ≈ 0.80). Quantitative titer correlation for high PRNT titers of ND_50_ ≥ 50, 200, and 1,000 was investigated with new cut-off values; the anti-RBD IgG antibody kit showed the best performance. It also showed the best linear correlation with PRNT titer in both the acute and convalescent phases (Pearson’s R 0.81 and 0.72, respectively). Due to the slowly waning titer of anti-NP antibodies, the correlation with PRNT titer at the convalescent phase was poor. In conclusion, semi-quantitative immunoassay kits targeting the RBD showed neutralizing activity that was correlated by titer; measurement of anti-NP antibodies would be useful for determining past infections.

## Introduction

Severe acute respiratory syndrome coronavirus 2 (SARS-CoV-2), causing coronavirus disease-19 (COVID-19), has caused more than 5 million deaths globally as of November 2021 (WHO, 2021). While novel vaccines for SARS-CoV-2 have helped control the pandemic, there are new variants capable of escaping immunogenicity acquired by natural infection and/or vaccination (Abdool Karim and de Oliveira, 2021; Gupta, 2021; Hacisuleyman et al., 2021). Waning of acquired immunity is a concern, and whether to administer a booster vaccine is another question that requires addressing (Baraniuk, 2021). Measurement of neutralization antibody levels is useful to predict protective immunity in patients who have recovered from COVID-19 and in those who have received vaccines (Khoury et al., 2021). However, neutralizing tests are usually not applicable in clinical laboratories because they require a biosafety level (BSL) 3 facility, skilled technicians, and considerable time for testing. To overcome these limitations, elaborate immunoassay kits applying various methodologies have been developed and have suggested a correlation with neutralization activities; however, the titer correlation with neutralization tests has not been elucidated. Herein, we evaluated the performance of semi-quantitative anti-SARS-CoV-2 spike protein antibody immunoassay kits in association with the titer of the neutralization test.

## Materials and Methods

### Study Population and Collected Specimens

Serum specimens were collected from three groups. First, serial serum specimens of patients with moderate-to-severe COVID-19 were collected from patients admitted to a tertiary care center (Ko et al., 2020a). Acute and convalescent specimens were collected, and sera collected after 14 days of illness were considered seroconverted-sera (Lau et al., 2021). Second, convalescent specimens from asymptomatic-to-mild COVID-19 were collected from patients staying at a residential care center at the time of discharge after two consecutive reverse transcriptase polymerase chain reaction (RT-PCR) tests (Ko et al., 2020a). Information about symptom onset and RT-PCR test results, including the cycle threshold (Ct) value, were retrospectively collected. Third, sera from healthcare workers (HCWs) collected before the spread of SARS-CoV-2 into the Korean community were used as negative control specimens. By the time of sampling, most COVID-19 cases in Korea could be epidemiologically traced, and none of the negative control patients had epidemiologic links to COVID-19 cases or the risk area. The absence of anti-SARS-CoV-2 antibodies in the negative control sera was confirmed by neutralization tests and/or multiple immunoassay kits (Ko et al., 2020b; Ko et al., 2021). While only 33 out of 126 sera form HCWs went through PRNT, the qualitative result of PRNT was imputed as negative for those without PRNT results. Written informed consent was obtained from each participant. The study was approved by the institutional review board (IRB) of each hospital (IRB No. SMC 2020-03-113, SMC 2020-04-006, SMC 2020-04-145, and KUMC 2020-07-067).

### Classification of Sera According to the Collection Time Point and Disease Severity

Specimens from the enrolled subjects with SARS-CoV-2 infection were reclassified by collection time points and disease severity. First, the sera were classified into either the acute phase specimens (collected before 21 days of illness) or convalescent phase specimens (collected since 21 days of illness), based on the time point of the peak serologic response of the present cohort. The baseline time point (day 0) was defined as follows: 1) if the patient was symptomatic before being diagnosed, the symptom onset was considered as the baseline, and 2) if the patient was asymptomatic at diagnosis, the date when the patient was diagnosed by RT-PCR was considered as the baseline. Second, for the classification of disease severity, COVID-19 patients were classified as “severe-to-critical” if the peak O_2_ requirement was greater than or equal to a fraction of inspired oxygen (FiO_2_) of 0.6. Otherwise, the patients were classified as “mild-to-moderate” cases.

### Plaque-Reduction Neutralization Test (PRNT)

PRNT was conducted at the Korea Disease Control and Prevention Agency (KDCA). Heat-inactivated (56°C for 30 min) serum samples were serially diluted four-fold with Dulbecco’s modified Eagle’s medium (DMEM) containing 2% fetal bovine serum (FBS) and 1% penicillin/streptomycin. Diluted serum was incubated at 37°C in a 5% CO_2_ incubator for 1 h. Fifty plaque-forming units (PFU)/well of SARS-CoV-2 (βCoV/Korea/KCDC03/2020 NCCP No.43326) were mixed with serum. The mixtures were inoculated into Vero E6 cells on a 24-well plate and incubated at 37°C and 5% CO_2_ for 1 h. After the inoculums were removed, the cells were overlaid with 1 ml of Minimum Essential Medium (MEM) containing 0.75% agarose and 2% FBS. The plates were incubated at 37°C in a 5% CO_2_ incubator for three days, following which the cells were stained with 0.07% crystal violet, 10% formaldehyde, and 5% ethanol, and the visualized plaques were counted. The 50% neutralizing dose (ND_50_) titer was calculated using the Kärber formula: log_10_ ND_50_ = m-Δ(∑p-0.5) (Grist et al., 1974).

### Surrogate Virus Neutralization Test (sVNT)

To detect neutralizing antibodies using an immunoassay method, the cPass sVNT kit (GenScript, Piscataway, NJ, USA) was used. sVNT measures the inhibition of interactions between the horseradish peroxidase (HRP)-conjugated SARS-CoV-2 spike protein receptor-binding domain (RBD) and the extracellular domain of the human angiotensin-converting enzyme 2 (hACE2) receptor (Taylor et al., 2021). The inhibition ratio is calculated as follows:


$$Inhibitionratio=\left(1-\frac{ODvalueofspecimen}{ODvalueofcontrol}\right)\times 100\%$$

The kit was approved as a qualitative test with a positive cut-off value of 30%, while the manufacturer suggested that semi-quantitative interpretation of the test would be possible (GenScript, 2020).

### Anti-SARS-CoV-2 Spike Protein Total Antibody Assay

To estimate total antibody titers against the RBD of the spike protein, the Elecsys^®^ Anti-SARS-CoV-2 S kit (Roche Diagnostics, Rotkreuz, Switzerland) was used. The kit was developed for *in vitro* qualitative and semi-quantitative measurement of anti-SARS-CoV-2 spike protein antibodies with an electro-chemiluminescence immunoassay (ECLIA) method using cobas e analyzers. A recombinant RBD of the spike protein was used with a double-antigen sandwich principle. While the antigen used in the kit was captured by IgG predominantly, IgA and IgM were detectable as well (Roche, 2020a). An anti-SARS-CoV-2 S antibody concentration ≥0.8 U/mL was considered positive. The linear range was 0.4–250 U/mL, and automated dilution was performed up to a 1:50 dilution in the cobas e analyzers. For results reported as <0.4, the values were imputed as 0.4.

### Anti-SARS-CoV-2 Spike Protein IgG Antibody Assay

The SARS-CoV-2 IgG II Quant kit ( Abbott Laboratories, Abbott Park, IL, USA) was used for the semi-quantitative measurement of IgG antibody titers against the RBD of the spike protein. The kit was developed for *in vitro* qualitative and semi-quantitative measurement of anti-SARS-CoV-2 spike protein IgG antibodies using a chemiluminescent microparticle immunoassay (CMIA) method using the Alinity and ARCHITECT Systems (Abbott, 2020b). Test results greater than or equal to 50.0 AU/mL were considered positive. The manufacturer suggests an analytic measuring interval (AMI) from 22.0 to 25,000.0 AU/mL, with acceptable performance for linearity. We performed automated 1:2 dilutions for the specimens with ≥25,000.0 AU/mL, as the manufacturer’s instruction suggests extending the measuring interval (EMI) from 25,000.0 to 50,000.0 AU/mL in 1:2 dilutions (Abbott, 2020a).

### Anti-SARS-CoV-2 Nucleocapsid Antibody Assay

To analyze the correlation between neutralization activity and anti-SARS-CoV-2 nucleocapsid antibody titers, Elecsys^®^ Anti-SARS-CoV-2 kit (Roche Diagnostics) was used. A recombinant nucleocapsid protein was used to detect high-affinity antibodies against SARS-CoV-2 (Muench et al., 2020). A double-antigen sandwich principle was utilized, and the ECLIA method was applied using cobas immunoassay analyzers. The detectable isotypes included IgA and IgG, and a cut-off index (COI) ≥1.0 was considered positive (Roche, 2020b). The kit was approved as a qualitative test, and the manufacturer did not suggest a titer correlation between the COI value and antibody titer. Nevertheless, the measured COI values were reported to be as high as 167 in the present analysis, and we investigated the correlation between COI values and the neutralizing titer of the study specimens.

### Statistical Analysis

The analytical performance for the neutralization activity of each immunoassay kit using the pre-defined cut-off value by the manufacturer was evaluated with sero-converted sera (from 14 days of illness) of confirmed COVID-19 patients as the positive group along with the negative control group. The performance was calculated for the prediction of PRNT ND_50_ values of ≥ 20, ≥ 40, and ≥ 80. Sensitivity, specificity, Cohen’s kappa, and area under the receiver operating characteristic curve (ROC AUC) values were calculated. The interpretation of Cohen’s kappa was as follows: values < 0.00 were considered as poor agreement, 0.00–0.20 as slight agreement, 0.21–0.40 as fair agreement, 0.41–0.60 as moderate agreement, 0.61–0.80 as substantial agreement, and 0.81–1.00 as almost perfect agreement (Landis and Koch, 1977).

Pearson’s correlation coefficient (R) and *P* values were calculated to investigate the titer correlation between each immunoassay kit and PRNT as a continuous variable. Subgroup analyses were conducted according to the pre-defined acute/convalescent phase. For the analysis of titer correlation as a categorical variable, we calculated the optimal cut-off values with the maximal Youden’s index for the prediction of PRNT ND_50_ values of ≥ 20, ≥ 50, ≥ 200, and ≥ 1,000. The analytical performance for each PRNT titer was analyzed based on the new cut-off values of the immunoassay kits. In order to compare the titers according to the timeline obtained with each assay, Wilcoxon’s test was performed.

The data were analyzed using Microsoft Excel (Microsoft, Redmond, WA, USA). Statistical analyses were performed with R 4.0.5 (R Foundation for Statistical Computing, Vienna, Austria). Sensitivity, specificity, and 95% intervals were calculated using the epiR 2.0.38 package on R 4.0.5. The plots were depicted with the ggplot2 3.3.3 and plotROC 2.2.1 packages on R 4.0.5.

## Results

### Characteristics of Study Population and Specimens

A total of 483 samples from 237 subjects were collected (**Table 1**). The median age of the subjects was 52 years (IQR 30–71 years), and the male-to-female ratio was 0.46. Among these, 357 samples from 111 patients were from COVID-19 patients with confirmed SARS-CoV-2 infection; 126 samples from 126 HCWs were used as negative controls. Specimens from the designated hospitals included 151 sera from the acute phase and 145 sera from the convalescent phase, and all specimens from the residential care center were collected at the convalescent phase. Eighty-nine patients at the residential care center and the designated hospitals experienced mild-to-moderate illness, while 19 patients at the designated hospitals progressed to severe-to-critical status.

**Table 1:** Characteristics of the study population and the specimens.

Variables	Total	COVID-19 patients		HCWs (Negative controls)
		Residential care center	Designated hospitals	
**Number of specimens** (patients)	483 (237)	61 (61)	296 (50)	126 (126)^* ‡^
**Age**, years	52.0 (30.0–71.0)	27.0 (24.0–37.0)	70.0 (61.0–74.0)	33.5 (27.0–42.0)
**Sex**, male: female	75:162	25:36	23:27	27:99
**Time point**				
Seroconverted (≥ 14 days)^†^	279 (110)	61 (61)	218 (49)	NA
Acute (< 21 days)^‡^	151 (41)	0 (0)	151 (41)	NA
Convalescent (≥ 21 days)^‡^	206 (101)	61 (61)	145 (40)	NA
**Severity of illness**				
Mild-to-moderate (FiO_2_ < 60%)	206 (89)	61 (61)	145 (28)	NA
Severe-to-critical (FiO_2_ ≥ 60%)	151 (22)	0 (0)	151 (22)	NA

Data are expressed as the number of specimens (patients) or as medians (IQR), unless indicated otherwise. ^*^While all the sera of COVID-19 patients underwent PRNT, 33 of 126 sera of HCWs underwent PRNT. ^†^For calculating the analytical performance in discriminating SARS-CoV-2 infection, sero-converted sera of COVID-19 patients were used as positive specimens and all the sera of HCWs were used as negative controls. ^‡^For the investigation of titer correlation between immunoassay kits and PRNT, all specimens of COVID-19 patients were used, and subgroup analyses were conducted according to the acute/convalescent phase.

COVID-19, coronavirus disease 2019; HCW, healthcare worker; FiO_2_, fraction of inspired oxygen; PRNT, plaque reduction neutralization test.

### Analytical Performance for the Discrimination of SARS-CoV-2 Infection

**Figure 1:**
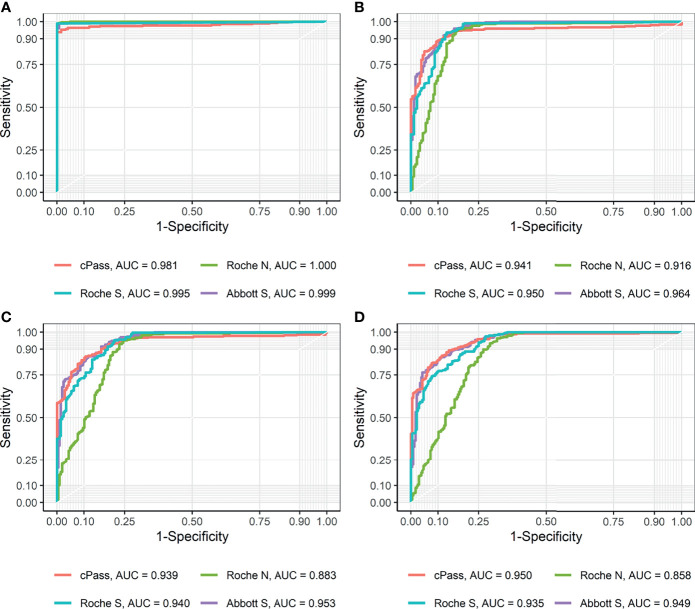
Receiver operating characteristic (ROC) curves and area under curve (AUC) for each method **(A)** using positive and negative controls and **(B–D)** compared to PRNT ND_50_ of (B) ≥ 20, (C) ≥ 40, and (D) ≥ 80, respectively.

The analytical performance of the discrimination of SARS-CoV-2 infection was evaluated using 279 sero-converted sera from 110 confirmed-COVID-19 patients and 126 negative control sera from 126 HCWs (**Table 2**). Both the Roche Elecsys Anti-SARS-CoV-2 and Roche Elecsys Anti-SARS-CoV-2 S kits demonstrated 100% specificity, which implies no false positive results in these two assays. All binding assays showed higher sensitivity and specificity than the cPass sVNT kit. The AUC values were all greater than 0.98, and cPass sVNT exhibited the lowest AUC of 0.981 (**Figure 1A**). The performance of each assay was comparable to that claimed by the manufacturer.

**Table 2:** Analytical performance of each kit in discriminating SARS-CoV-2 infection.

Kit, manufacturer, target protein, and Ab measured	Performance		Kappa
	Sensitivity (95% CI)	Specificity (95% CI)	
**cPass sVNT**, *GenScript, RBD, total*	96.42% (93.51%–98.27%)	95.24% (89.92%–98.23%)	0.91
**Elecsys Anti-SARS-CoV-2**, *Roche, NP, IgG/IgA*	98.92% (96.89%–99.78%)	100.00% (97.11%–100.00%)	0.98
**Elecsys Anti-SARS-CoV-2 S**, *Roche, RBD, total*	98.21% (95.87%–99.42%)	100.00% (97.11%–100.00%)	0.97
**SARS-CoV-2 IgG II Quant**, *Abbott, RBD, IgG*	98.92% (96.89%–99.78%)	99.21% (95.66%–99.98%)	0.98

For calculating the analytical performance in discriminating SARS-CoV-2 infection, 279 sero-converted sera of confirmed-COVID-19 patients and 126 negative control sera of HCWs were used. The pre-defined cut-off values suggested by the manufacturers were applied.

SARS-CoV-2, severe acute respiratory syndrome coronavirus 2; Ab, antibody; sVNT, surrogate virus neutralization test; RBD, receptor-binding domain; NP, nucleocapsid; CI, confidence interval.

### Analytical Performance for Representativeness of Neutralizing Activity Using Pre-Defined Cut-Off Values

The analytical performance in terms of the representation of the neutralizing activity, with PRNT cut-off values of ND_50_ ≥ 20, ≥ 40, and ≥ 80, was evaluated using 357 acute and convalescent sera of 111 confirmed-COVID-19 patients and 40 negative control sera from 40 HCWs (**Table 3**). For each immunoassay kit, pre-defined cut-off values suggested by the manufacturers were applied. When compared with ND_50_ ≥ 20, which is the cut-off commonly used for designating the presence of neutralization activity, all methods exhibited results highly concordant with PRNT, with a Cohen’s kappa of approximately 0.80. The Abbott SARS-CoV-2 IgG II Quant kit demonstrated a Cohen’s kappa of 0.81, showing a substantial agreement with PRNT, which was the highest value among the tested kits. The RBD-targeting semi-quantitative kits showed high sensitivity: GenScript cPass sVNT (94.68%), Roche Elecsys Anti-SARS-CoV-2 S (96.68%), and Abbott SARS-CoV-2 IgG II Quant (97.67%). While the Roche Elecsys Anti-SARS-CoV 2 assay, the only assay that targets NP in this study, showed lower sensitivity (94.35%) compared to other assays, its specificity (83.52%) was the highest among the assays compared. When pre-defined cutoffs provided by the manufacturers were applied, the ability to predict the neutralization effect (Cohen’s kappa) declined for all assays as the cut-off for ND_50_ increased. While a strong agreement with ND_50_ ≥ 20 was observed for each method, the need for a different cut-off to predict a high titer of neutralization effect was raised.

**Table 3:** Analytical performance for representativeness of neutralizing activity using the pre-defined cut-off values of each immunoassay kit.

PRNT cut-off	Kit, manufacturer, target protein, and Ab measured	Performance		
		Sensitivity (95% CI)	Specificity (95% CI)	Kappa
**ND_50_ ≥ 20**	**cPass sVNT**, *GenScript, RBD, total*	94.68% (91.51%–96.93%)	81.32% (74.89%–86.70%)	0.78
	**Elecsys Anti-SARS-CoV-2**, *Roche, NP, IgG/IgA*	94.35% (91.11%–96.68%)	83.52% (77.31%–88.59%)	0.79
	**Elecsys Anti-SARS-CoV-2 S**, *Roche, RBD, total*	96.68% (94.40%–98.62%)	80.77% (74.28%–86.22%)	0.80
	**SARS-CoV-2 IgG II Quant**, *Abbott, RBD, IgG*	97.67% (95.25%–99.06%)	80.77% (74.28%–86.22%)	0.81
**ND_50_ ≥ 40**	**cPass sVNT**, *GenScript, RBD, total*	96.00% (92.96%–97.99%)	73.56% (67.01%–79.42%)	0.71
	**Elecsys Anti-SARS-CoV-2**, *Roche, NP, IgG/IgA*	95.27% (92.05%–97.46%)	75.00% (68.54%–80.73%)	0.72
	**Elecsys Anti-SARS-CoV-2 S**, *Roche, RBD, total*	98.18% (95.81%–99.41%)	72.60% (66.00%–78.54%)	0.73
	**SARS-CoV-2 IgG II Quant**, *Abbott, RBD, IgG*	98.18% (95.79%–99.40%)	71.63% (64.99%–77.65%)	0.72
**ND_50_ ≥ 80**	**cPass sVNT**, *GenScript, RBD, total*	98.38% (95.91%–99.56%)	67.80% (61.43%–73.71%)	0.67
	**Elecsys Anti-SARS-CoV-2**, *Roche, NP, IgG/IgA*	96.36% (93.20%–98.32%)	67.80% (61.43%–73.71%)	0.65
	**Elecsys Anti-SARS-CoV-2 S**, *Roche, RBD, total*	99.19% (97.11%–99.90%)	65.25% (58.80%–71.31%)	0.65
	**SARS-CoV-2 IgG II Quant**, *Abbott, RBD, IgG*	99.59% (97.76%–99.99%)	64.83% (58.37%–70.91%)	0.65

For calculating the analytical performance in discriminating SARS-CoV-2 infection, 357 sero-converted sera of confirmed-COVID-19 patients and 126 negative control sera of HCWs were used. The pre-defined cut-off values suggested by the manufacturers were applied.

SARS-CoV-2, severe acute respiratory syndrome coronavirus 2; sVNT, surrogate virus neutralization test; RBD, receptor-binding domain; NP, nucleocapsid; CI, confidence interval.

### Correlation With PRNT Titers and Corresponsive New Cut-Off Values

The ROC AUCs for the prediction of PRNT ND_50_ ≥ 20 of the GenScript cPass sVNT, Roche Elecsys Anti-SARS-CoV-2, Roche Elecsys Anti-SARS-CoV-2 S, and Abbott SARS-CoV-2 IgG II Quant kits were 0.941, 0.916, 0.950, and 0.964, respectively (**Figure 1B**). Based on the Youden’s index found in the ROC curve, new cut-offs that represent the neutralizing activity of PRNT ND_50_ ≥ 20 were established. The cut-offs that best predict the neutralization activity of PRNT ND_50_ ≥ 20 were higher than the pre-defined cut-offs of the GenScript cPass sVNT (new value of 39.65% and pre-defined value of 30.0%), Roche Elecsys Anti-SARS-CoV-2 S (new value of 4.08 U/mL and pre-defined value of 0.8 U/mL), and Abbott SARS-CoV-2 IgG II Quant kits (new value of 120.1 AU/mL and pre-defined value of 50.0 AU/mL). For the Roche Elecsys Anti-SARS-CoV-2 assay, the new cut-off value was lower than the pre-defined cut-off value provided by the manufacturer (new value of 0.65 COI and pre-defined value of 1.0 COI), which showed higher sensitivity by sacrificing specificity. For other methods targeting the RBD, the new cut-offs achieved higher specificity at the cost of lower sensitivity compared to the pre-defined cut-offs. There were no significant differences in Cohen’s kappa.

To investigate new cut-off values representing higher neutralizing antibody titers and analytic performances, Youden’s indices in the ROC curve were utilized in the same manner to establish cut-offs for each assay that best represented ND_50_ values ≥ 50, ≥ 200, and ≥ 1,000 (**Table 4** and **Appendix** **Figure 1**). While there was no significant difference between the pre-defined cut-off and the new cut-offs for the Roche Elecsys Anti-SARS-CoV-2 targeting the nucleocapsid protein, the new cut-offs of assays targeting the RBD increased with higher PRNT cut-offs. As the target PRNT titer increased, Cohen’s kappa declined, despite adopting the new cut-offs derived from Youden’s indices. According to the new cut-offs, the Abbott SARS-CoV-2 IgG II Quant kit demonstrated the highest agreement in predicting high titers of neutralizing antibodies, followed by the GenScript cPass sVNT and Roche Elecsys Anti-SARS-CoV-2 S kits. Binding assays targeting the RBD demonstrated results comparable with GenScript cPass sVNT. However, the Roche Elecsys Anti-SARS-CoV-2 assay showed significantly lower Cohen’s kappa compared to other assays, implying its limited use in predicting high neutralization activity.

**Table 4:** Titer correlation of the analytical performance of the prediction of neutralizing activity using newly calculated cut-off values determined using Youden’s index.

PRNT titers	Kit, Manufacturer, target protein, Ab measured, and pre-defined cut-off	New cut-off values	Performance		
			Sensitivity (95% CI)	Specificity (95% CI)	Kappa
**ND_50_** **≥ 20**	**cPass sVNT**, *GenScript, RBD, total, 30%*	39.65%	91.03% (87.22%–94.01%)	87.91% (82.27%–92.27%)	0.79
	**Elecsys Anti-SARS-CoV-2**, *Roche, NP, IgG/IgA, 1.0 COI*	0.65 COI	95.68% (92.73%–97.68%)	82.42% (76.10%–87.65%)	0.80
	**Elecsys Anti-SARS-CoV-2 S**, *Roche, RBD, total, 1.0 U/mL*	4.08 U/mL	93.69% (90.32%–96.16%)	86.81% (81.02%–91.36%)	0.81
	**SARS-CoV-2 IgG II Quant**, *Abbott, RBD, IgG, 50 AU/mL*	120.1 AU/mL	96.00% (93.12%–97.92%)	84.07% (77.92%–89.06%)	0.82
**ND_50_** **≥ 50**	**cPass sVNT**, *GenScript, RBD, total, 30%*	59.7%	85.77% (80.99%–89.73%)	88.89% (83.92%–92.75%)	0.74
	**Elecsys Anti-SARS-CoV-2**, *Roche, NP, IgG/IgA, 1.0 COI*	1.1 COI	95.51% (92.28%–97.66%)	73.15% (66.71%–78.93%)	0.70
	**Elecsys Anti-SARS-CoV-2 S**, *Roche, RBD, total, 1.0 U/mL*	4.1 U/mL	94.76% (91.36%–97.10%)	76.39% (70.15%–81.89%)	0.72
	**SARS-CoV-2 IgG II Quant**, *Abbott, RBD, IgG, 50 AU/mL*	449.7 AU/mL	87.22% (82.60%–90.98%)	87.50% (82.34%–91.60%)	0.74
**ND_50_** **≥ 200**	**cPass sVNT**, *GenScript, RBD, total, 30%*	61.7%	93.43% (89.03%–96.46%)	77.89% (72.62%–82.58%)	0.69
	**Elecsys Anti-SARS-CoV-2**, *Roche, NP, IgG/IgA, 1.0 COI*	1.1 COI	97.47% (94.21%–99.18%)	57.89% (51.93%–63.69%)	0.51
	**Elecsys Anti-SARS-CoV-2 S**, *Roche, RBD, total, 1.0 U/mL*	60.6 U/mL	83.84% (77.96%–88.68%)	82.81% (77.92%–87.00%)	0.66
	**SARS-CoV-2 IgG II Quant**, *Abbott, RBD, IgG, 50 AU/mL*	1665.3 AU/mL	86.29% (80.69%–90.77%)	90.53% (86.52%–93.66%)	0.77
**ND_50_** **≥ 1000**	**cPass sVNT**, *GenScript, RBD, total, 30%*	86.7%	83.84% (75.09%–90.47%)	80.47% (76.14%–84.32%)	0.53
	**Elecsys Anti-SARS-CoV-2**, *Roche, NP, IgG/IgA, 1.0 COI*	1.2 COI	100.00% (96.34%–100.00%)	44.79% (39.74%–49.92%)	0.25
	**Elecsys Anti-SARS-CoV-2 S**, *Roche, RBD, total, 1.0 U/mL*	127.0 U/mL	87.88% (79.78%–93.58%)	76.30% (71.73%–80.47%)	0.50
	**SARS-CoV-2 IgG II Quant**, *Abbott, RBD, IgG, 50 AU/mL*	2836.2 AU/mL	97.96% (92.82%–99.75%)	79.69% (75.31%–83.60%)	0.60

For calculating the analytical performance in discriminating SARS-CoV-2 infection, 357 sero-converted sera of confirmed-COVID-19 patients and 126 negative control sera of HCWs were used. The new cut-off value for each kit was calculated using the Youden’s index.

SARS-CoV-2, severe acute respiratory syndrome coronavirus 2; sVNT, surrogate virus neutralization test; RBD, receptor-binding domain; NP, nucleocapsid; CI, confidence interval.

### Serial Kinetics of PRNT Titers and Semi-Quantitative Immunoassay Kits

**Figure 2:**
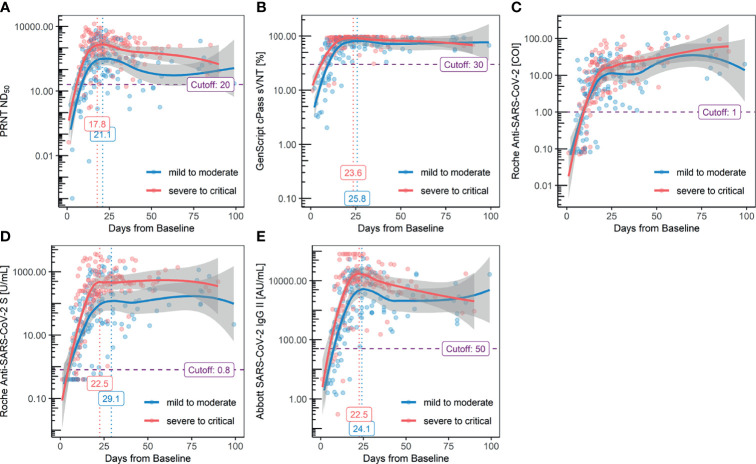
Serial kinetics of antibody titers measured with each method: **(A)** PRNT ND50, **(B.)** GenScript cPass sVNT, **(C)** Roche Elecsys Anti-SARS-CoV-2, **(D)** Roche Elecsys Anti-SARS-CoV2 S, and **(E)** Abbott AdviseDx SARS-CoV2 IgG II.

For the categorization of the acute and convalescent phases, serial kinetics of each antibody assay were plotted using positive samples and divided by disease severity (**Figure 2**). According to the results of PRNT of positive samples, seroconversion was observed at 5.2 days from baseline, and peak titer was observed at 18.7 days from baseline. When this group was divided by severity, seroconversion was 6.2 days from baseline in the mild-to-moderate group (FiO2 ≤ 0.6) and 3.9 days from baseline in the severe-to-critical group (FiO2 > 0.6).

The peak titer was reached after 19.4 days from baseline in the mild-to-moderate group and 18.0 days in the severe-to-critical group. Compared to the kits targeting the RBD exhibiting a descending trend after reaching the peak titer at approximately 2–3 weeks, the results of the Roche Elecsys Anti-SARS-CoV-2 kit consistently increased, even after 3 weeks from the baseline. While the serial kinetics of the GenScript cPass sVNT kit was in line with other assays targeting the RBD, a decline in antibody titer after reaching the peak was not evident owing to early saturation of the method, regardless of the severity. Higher antibody titers in the severe-to-critical group were observed with the Roche Elecsys Anti-SARS-CoV-2 S and Abbott SARS-CoV-2 IgG II kits. The Abbott SARS-CoV-2 IgG II Quant kit revealed the waning of antibody titer prominently compared to the Roche Elecsys Anti-SARS-CoV-2 S assay. The antibody titers, measured with each assay, were categorized into four groups by timeline: 1) 1^st^ week (1–6 days, before seroconversion), 2) 2^nd^–3^rd^ weeks (7–13 days, acute rising), 3) 3^rd^–4^th^ weeks (14–27 days, peak titers), and 4) 5^th^–15^th^ weeks (28–104 days, waning titers); the results are summarized in **Table 5**. A statistically significant decrease in antibody titer after reaching the peak was observed for all kits targeting the RBD, except for the Roche Elecsys Anti-SARS-CoV-2 S kit.

**Table 5:** Antibody titers by timeline.

Kit, manufacturer, target, and Ab measured	1^st^ week (1–6 days)* before seroconversion*		2^nd^ to 3^rd^ weeks (7–13 days) *acute rising*		3^rd^ to 4^th^ weeks (14–27 days) *peak titer*		5^th^ to 15^th^ weeks (28–104 days) *waning titer*	
**PRNT ND_50_** *KDCA, SARS-CoV-2, total*	7.71 ± 9.68		938.57 ± 1889.93		1705.73 ± 2126.20		597.05 ± 870.22	
		┕ ***P* < 0.001** ┙		┕ ***P* < 0.001** ┙		┕ ***P* < 0.001** ┙		
**cPass sVNT**, *GenScript, RBD, total*	13.57 ± 15.64%		46.81 ± 32.54%		83.08 ± 17.00%		74.53 ± 22.87%	
		┕ ***P* < 0.001** ┙		┕ ***P* < 0.001** ┙		┕ ***P* < 0.01** ┙		
**Elecsys Anti-SARS-CoV-2**, *Roche, NP, IgG/IgA*	2.17 ± 7.07 COI		3.48 ± 5.07 COI		15.62 ± 15.00 COI		36.30 ± 33.57 COI	
		┕ ***P* < 0.01** ┙		┕ ***P* < 0.001** ┙		┕ ***P* < 0.001** ┙		
**Elecsys Anti-SARS-CoV-2 S**, *Roche, RBD, total*	6.16 ± 19.88 U/mL		97.18 ± 321.84 U/mL		486.81 ± 745.27 U/mL		373.05 ± 502.61 U/mL	
		┕ ***P* < 0.001** ┙		┕ ***P* < 0.001** ┙		┕ *P* = 0.9394 ┙		
**SARS-CoV-2 IgG II Quant**, *Abbott, RBD, IgG*	34.65 ± 57.75 AU/mL		5337.75 ± 15426.61 AU/mL		16806.08 ± 21912.72 AU/mL		5959.90 ± 8336.19 AU/mL	
		┕ ***P* < 0.001** ┙		┕ ***P* < 0.001** ┙		┕ ***P* < 0.001** ┙		

Ab, antibody; SARS-CoV-2, severe acute respiratory syndrome coronavirus 2; ND_50_, 50% neutralizing dose; KDCA, Korea Disease Control and Prevention Agency; sVNT, surrogate virus neutralization test; RBD, receptor-binding domain; NP, nucleocapsid.

The bold values indicate those with statistical significance.

### Linear Correlation Between PRNT Titers and Semi-Quantitative Immunoassay Values

**Figure 3:**
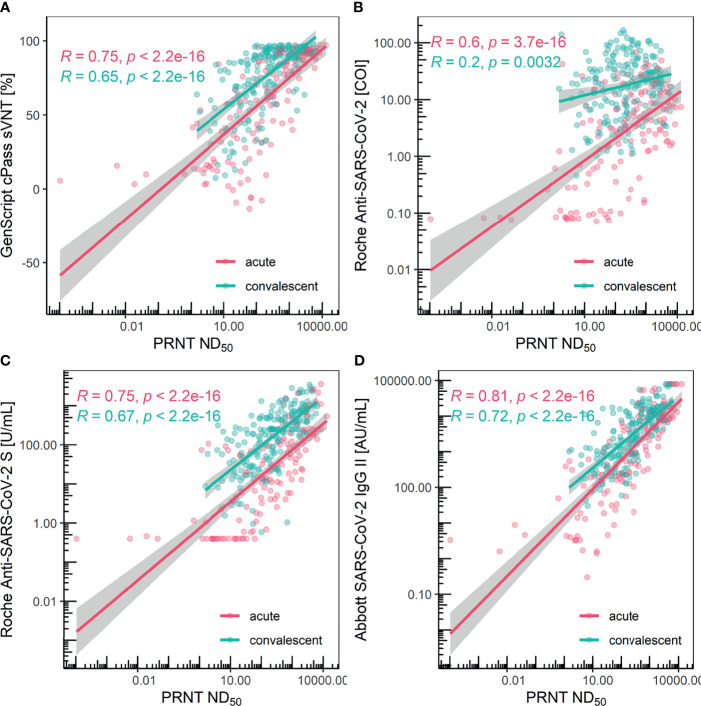
Scatter plot and Pearson’s correlation for each method grouped with acute/convalescent phase. **(A)** GenScript cPass sVNT, **(B)** Roche Elecsys Anti-SARS-CoV-2, **(C)** Roche Elecsys Anti-SARS-CoV2 S, and **(D)** Abbott AdviseDx SARS-CoV2 IgG II were compared with PRNT, respectively. Each colored line depicts the linear regression model and the surrounding grey-colored area represents the 95% confidence interval.

The assays showed significantly different correlation results when compared with PRNT. Pearson’s correlation coefficients for the GenScript cPass sVNT kit were 0.75 and 0.65 for the acute and convalescent phase, respectively. Pearson’s correlation coefficients for the Roche Elecsys Anti-SARS-CoV-2 kit were 0.60 and 0.20 for the acute and convalescent phase, respectively. Pearson’s correlation coefficients for the Roche Elecsys Anti-SARS-CoV-2 S kit were 0.75 and 0.67 for the acute and convalescent phase, respectively. Pearson’s correlation coefficients for the Abbott AdviseDx SARS-CoV-2 IgG II kit were 0.81 and 0.72 for the acute and convalescent phases, respectively, the highest among the compared assays. All comparisons showed statistically significant *P* values (**Figure 3**).

## Discussion

The situation surrounding the COVID-19 pandemic has changed drastically over the last two years. Multiple serologic tests for SARS-CoV-2 have been developed and used for various indications such as: diagnosing recent or past infections, performing sero-prevalence studies assessing herd immunity, sero-epidemiologic tracing of outbreak clusters, and risk assessment of healthcare workers; preparing convalescence plasma (CP) therapy, assessing neutralizing antibodies in COVID-19 patients, and evaluating protective immunity from past infections and/or vaccinations; according to the status of the COVID-19 pandemic, the significance of each clinical implication has differed (Ko et al., 2017a; Ko et al., 2017b; Ko et al., 2017c; Ko et al., 2018; Ahn et al., 2020; Ko et al., 2020a; Ko et al., 2020b; Park et al., 2020; Yong et al., 2020; Khoury et al., 2021; Lau et al., 2021; van Kampen et al., 2021). Among these clinical implications, while the presence of binding antibodies is important for seroprevalence studies to distinguish previous infections, the detection and quantification of neutralizing antibodies are crucial for several indications, including the preparation of CP therapy, assessment of neutralizing antibodies in COVID-19 patients, and evaluation of protective immunity. The clinical utility of immunoassays would be substantiated if the magnitude of neutralization can be estimated by using it in routine clinical practice. Hence, for appropriate clinical application of serologic studies, the importance of titer correlation evaluation cannot be overemphasized.

In this study, three semi-quantitative assays targeting the RBD demonstrated a linear correlation with the neutralizing antibody titer measured using PRNT. In terms of performance in predicting the neutralization titer, the Abbott SARS-CoV-2 IgG II Quant kit, an IgG-specific binding assay, was the best, followed by other assays measuring antibody titers regardless of immunoglobulin isotypes: GenScript cPass sVNT and Roche Elecsys Anti-SARS-CoV-2 S. This could be due to IgG consisting of the majority of the antibody isotypes that target the RBD (Klein et al., 2020) and due to the different isotypes showing different epitope repertoires within the RBD (Tang et al., 2021). Moreover, the measurement techniques utilized and the measurement range in each assay may have affected the performance. Up to a two-fold dilution was performed for the Abbott AdviseDx SARS-CoV2 IgG II Quant assay, which uses the CMIA method; up to 50-fold dilution was carried out for the Roche Elecsys Anti-SARS-CoV-2 S assay, which uses the ECLIA method due to its narrow measurement range with early saturation. For the GenScript cPass sVNT kit, dilution was not conducted because the method represents the result of the inhibition ratio calculated from the optical density measured, which does not necessarily linearly correlate with the titer of the antibodies.

Although GenScript cPass sVNT kit exhibited a lower Pearson’s correlation coefficient than the Abbott AdviseDx SARS-CoV-2 IgG II, this may be due to the narrower reportable range of GenScript cPass sVNT, leading to early saturation in subjects with high antibody titers. Although it is true that GenScript cPass sVNT stands in a disadvantageous position since serial dilution was performed for results exceeding the reportable range in binding assays, while this did not occur in GenScript cPass sVNT, it is noteworthy that GenScript cPass sVNT is relatively more time-consuming and labor-intensive compared to binding assays. The GenScript cPass sVNT kit utilizes the inhibition of binding with the ACE2 receptor and this technique suggests superiority as the result itself is a surrogate of the neutralization activity. However, for a more accurate assessment of high titers with GenScript cPass sVNT, further investigation of how the inhibition ratio changes with dilutions is required as different trends in results following dilution have been reported depending on the composition of immunoglobulin isotypes (Tan et al., 2020).

There has been a study using monoclonal antibodies where sVNT was able to differentiate between neutralizing antibodies and binding antibodies, while ELISA using the identical RBD antigen failed to distinguish neutralizing antibodies (Tan et al., 2020). Although a number of serological assays utilize the RBD as the antigenic target, which is the same as for GenScript cPass sVNT, the protein coating process can cause exposure of hidden epitopes and changes in epitopes that do not exist in the natural state, which occur due to conformational changes (Lee and Belfort, 1989; Sethuraman and Belfort, 2005; Raffaini and Ganazzoli, 2010; Guven et al., 2014; de Thier et al., 2015). This phenomenon could result in lower specificity due to antibodies binding to newly appearing epitopes (Mannik et al., 1997; Guven et al., 2014).

However, despite this limitation of serological assays, a high correlation between anti-RBD IgG and neutralizing antibodies has been shown in previous studies (Dispinseri et al., 2021; Dogan et al., 2021), and our study shows the feasibility of using semiquantitative serologic assays targeting the RBD in predicting the neutralization titer with Abbott AdviseDx SARS-CoV2 IgG II, which measures IgG against the RBD showing the highest performance. In real-world clinical practice, there is little chance of mutually exclusive presence of binding antibodies and neutralizing antibodies. As SARS-CoV-2 infection occurs, the immune system exhibits avidity maturation over time (Luo et al., 2020); patient samples are a complex mixture of antibodies with different binding affinities. Therefore, although separated monoclonal antibodies exhibit discordant results between sVNT and binding assays (Tan et al., 2020), binding assays may be used to estimate neutralization activity in clinical practice.

It is noteworthy that there were significant differences between the acute phase and the convalescent phase in titer correlation with PRNT. During the acute phase, all assays showed a fair correlation with PRNT, since antibodies with different isotypes targeting various epitopes exhibit a rising trend. However, in the convalescent phase, as antibody affinity maturation and titer waning occur, the difference in the correlation with PRNT of different kits becomes evident. Although the Roche Elecsys Anti-SARS-CoV-2 kit measuring nucleocapsid antibodies correlated with PRNT during the acute phase, this is primarily due to the nucleocapsid protein being the most abundant viral antigen in the early stages of infection (Satarker and Nampoothiri, 2020; Chura-Chambi et al., 2022). The poor correlation between the Roche Elecsys Anti-SARS-CoV-2 and PRNT during the convalescent phase implies that nucleocapsid antibodies are not suitable for predicting the neutralization titer. Thus, it is suggested that convalescent sera be used to evaluate the performance in predicting the neutralization titer measured with PRNT.

While the Roche Elecsys Anti-SARS-CoV-2 kit, which measures the antibodies targeting the nucleocapsid protein, performed poorly in predicting the neutralization titer, it showed the highest sensitivity and specificity in determining the diagnosis of SARS-CoV-2 infection. Furthermore, although both PRNT and the assays targeting the RBD showed a declining trend during the convalescent phase, a persistently high value was observed with the Roche Elecsys Anti-SARS-CoV-2 kit, though the Roche Elecsys Anti-SARS-CoV-2 kit has been approved as a qualitative assay. We suggest that measuring nucleocapsid antibodies would be beneficial in seroprevalence studies in order to identify past infections and in determining breakthrough infection in vaccinated populations as well. In conclusion, the distinct characteristics of nucleocapsid antibodies compared to RBD antibodies highlight the clinical significance of measuring nucleocapsid antibodies as the vaccination rate increases.

Our study has several limitations. First, the number of acute phase samples and subjects was relatively small compared to the those in the convalescent phase because only convalescent samples were collected from patients managed at the residential care center. However, our study was able to address the analytical performance and correlation of each assay using samples with a wide range of antibody concentrations. Additionally, because whether the antibodies that last in the convalescent phase are binding antibodies or neutralizing antibodies remains unknown, the performance of each assay in the convalescent phase is a major concern. Second, although a number of samples were collected for the “severe-to-critical” group, the limited number of patients may not fully represent the disease spectrum. Patients with different clinical courses can exhibit distinguishing antibody kinetics. For instance, there could be cases where low-affinity binding antibodies persist despite the rapid waning of neutralizing antibodies, resulting in discordant results between binding assays and neutralization tests. Thus, further research should be conducted to address these limitations. In addition, the assays analyzed in this study were developed before the appearance of the new variant. Since antibodies against different variants show different affinities against the recombinant RBD used in each assay, re-validation of the assays is warranted for suitability in the current situation. Furthermore, the clinical utility of binding assays and the ability to represent neutralization activity should be assessed for vaccinees.

In summary, our study illustrates the utility of immunoassays against the RBD of SARS-CoV-2 in predicting neutralization activity. While measuring anti-NP antibodies demonstrated the best performance in determining past infections, the semi-quantitative assays targeting the RBD demonstrated linearly correlated results with PRNT, and the measurement of IgG was thought to be crucial in estimating neutralizing antibodies compared to immunoglobulins of other isotypes.

## Data Availability Statement

The original contributions presented in the study are included in the article/supplementary material. Further inquiries can be directed to the corresponding authors.

## Ethics Statement

The study was approved by the institutional review board (IRB) of each hospital (IRB No. SMC 2020-03-113, SMC 2020-04-006, SMC 2020-04-145, and KUMC 2020-07-067). Written informed consent to participate in this study was provided by the participants’ legal guardian/next of kin.

## Author Contributions

Conceptualization, BL, J-HK, JP, E-JJ, E-SK, and KP. Investigation, BL, J-HK, JP, H-WM, JB, SJ, H-YL, K-CK, KH, SC, C-IK, DC, HH, CC, Y-JK, and E-JJ. Laboratory work, JB, SJ, H-YL, K-CK, and E-SK. Data analysis, BL and J-HK. Writing–review and editing, BL, J-HK, E-JJ, E-SK, and KP. All authors have read and agreed to the manuscript. All authors contributed to the article and approved the submitted version.

## Funding

This work was supported by Research Program funded by the Korea Disease Control and Prevention Agency (#2020-ER5328-00 and #2020-NI-013-00) and Samsung Medical Center Grant (#SMO1210321).

## Conflict of Interest

The authors declare that the research was conducted in the absence of any commercial or financial relationships that could be construed as a potential conflict of interest.

## Publisher’s Note

All claims expressed in this article are solely those of the authors and do not necessarily represent those of their affiliated organizations, or those of the publisher, the editors and the reviewers. Any product that may be evaluated in this article, or claim that may be made by its manufacturer, is not guaranteed or endorsed by the publisher.
